# Sex-based differences in the long-term fate of hippocampal neurons born after a traumatic brain injury

**DOI:** 10.3389/fnbeh.2025.1523969

**Published:** 2025-02-05

**Authors:** Hannah C. Downing, Ashley B. Glover, Jessica E. Gebhardt, Katherine L. Thompson, Kathryn E. Saatman

**Affiliations:** ^1^Department of Physiology, University of Kentucky, Lexington, KY, United States; ^2^Spinal Cord and Brain Injury Research Center, University of Kentucky, Lexington, KY, United States; ^3^Department of Neuroscience, University of Kentucky, Lexington, KY, United States; ^4^Dr. Bing Zhang Department of Statistics, College of Arts and Sciences, University of Kentucky, Lexington, KY, United States

**Keywords:** dendrite morphology, dentate gyrus, hippocampus, mossy fiber boutons, neurogenesis, sex differences, synapses, traumatic brain injury

## Abstract

**Introduction:**

Moderate-to-severe traumatic brain injury (TBI) results in an early loss of immature hippocampal granule cells and the activation of typically quiescent neural stem cells (NSCs) in the dentate gyrus. Activation of NSCs leads to a robust increase in proliferation and generation of neural progenitor cells (NPCs), supporting restoration of the immature neuron population of over a period of 1–2 weeks. However, it is unclear if neurons born early after injury develop normally, survive long-term and functionally integrate into the hippocampal network. Although adult hippocampal neurogenesis is regulated in a sex-dependent manner, the majority of pre-clinical TBI studies lack the inclusion of both sexes. The goal of this study was to examine sex differences in hippocampal neurogenesis in response to a moderate controlled cortical impact brain injury.

**Methods:**

*In-vivo* labeling of NPCs and tracking of their morphological development into a granule cell was achieved using an inducible Cre recombinase driven by the Ascl1 promoter in a CAG-floxStopTom reporter mouse. Ascl1 is a basic helix-loop-helix transcription factor transiently expressed in NPCs and activated NSCs in the dentate gyrus of the adult mammalian brain. To specifically label NPCs born acutely after TBI, tamoxifen was delivered to mice on days 2 and 3 postinjury. Mice survived to 6 weeks after TBI to allow for full neuronal maturation of tdTomato-labeled NPCs.

**Results:**

At 6 weeks postinjury, numbers of tdTomato-positive granule cells were significantly reduced in the ipsilateral hippocampus of brain-injured mice compared to controls, with a more pronounced decrease in males. Further, posttrauma-born neurons in males, but not females, exhibited impaired dendritic development. Neurons born after injury extended axons which formed synaptic terminals within the CA3 region. Numbers of mossy fiber boutons were significantly decreased in injured males compared to naïve males or to injured females. Potential forms of plasticity were observed in brain-injured females, including increased neurogenesis in the contralateral hippocampus and increased mossy fiber bouton volume. Together these data suggest a neurogenic advantage in females after injury.

**Discussion:**

This study is the first to report sex differences in posttraumatic hippocampal neurogenesis and to demonstrate modification of synaptic terminals formed by neurons born after TBI.

## 1 Introduction

More than 5.3 million Americans are currently living with a disability related to traumatic brain injury (TBI). Over half of individuals with moderate to severe TBI report long-term problems such as executive function and cognitive deficits that hinder their ability to return to work or school (Rabinowitz and Levin, 2014), with cognitive dysfunction being the primary source of TBI-related disability (CDC, 2015). The hippocampus, a key neuroanatomical structure involved in learning and memory, is structurally and functionally vulnerable to TBI. Concomitant with the early loss of immature neurons in the dentate gyrus (DG), hippocampal neural stem cells (NSCs) located in the subgranular zone (SGZ) of the DG become activated and robustly increase their rate of cellular proliferation to generate neural progenitor cells (NPCs). Numbers of proliferating cells in the SGZ significantly increase at 2–3 days after injury and return to baseline levels by 2 weeks (Dash et al., 2001; Gao and Chen, 2013; Rola et al., 2006). These cells have the potential to develop into granule cells (GCs), replenishing the immature GC population and supporting cognitive recovery (Yu et al., 2008; Blaiss et al., 2011; Sun et al., 2015). Cells birth-dated in the acute postinjury period through DNA incorporation of bromodeoxyuridine (BrdU) during proliferation can be detected in the GC layer 1 month or longer after injury. While many proliferated cells in the injured hippocampus phenotype as glial cells, co-labeling with mature neuron markers demonstrates that a subset of posttrauma-born neurons survive to reach maturity (Dash et al., 2001; Gao and Chen, 2013; Littlejohn et al., 2020).

Adult-generated GCs take 3–4 weeks to fully mature (van Praag et al., 2002). Once progenitor cells exit the cell cycle and differentiate into a post-mitotic immature neuron, they begin their dendritic development and extend axonal projections toward the CA3 region of the hippocampus (Brandt et al., 2003; Plumpe et al., 2006). Axonal projections of newborn GCs can be detected within 2 weeks (Faulkner et al., 2008; Toni et al., 2008). By 4 weeks new adult-born neurons have established their final position in the inner and middle layers of the GC layer and have elaborated spiny dendrites reaching into the outer molecular layer (Esposito et al., 2005).

Multiple aspects of maturation of adult-born neurons are dysregulated by traumatic injury. Following TBI, posttrauma-born neurons exhibit abnormal dendritic development at both immature and mature phases and a subpopulation migrate beyond their typical positioning, to the outer layers of the GC layer (Carlson et al., 2014; Ibrahim et al., 2016; Littlejohn et al., 2020; Shahror et al., 2020; Villasana et al., 2015; Zhang et al., 2020). However, little known about the fidelity of axonal projections elaborated by neurons born after TBI. Two studies demonstrated using retrograde labeling that GCs born after TBI extend axons to the CA3 pyramidal region (Emery et al., 2005; Sun et al., 2007). Axons of GCs comprise the mossy fiber pathway and have three morphologically and functionally distinct presynaptic terminals: small en passant boutons, large mossy fiber boutons (MFBs), and filopodial extensions that emerge from the MFBs (Rollenhagen and Lubke, 2010). Changes in MFB numbers and size alter connectivity of GCs with CA3 pyramidal neurons thereby influencing hippocampal function. Formation, persistence and size of MFBs associated with adult-born GCs are modulated by age (Murray et al., 2020), but how TBI affects MFB formation by axons developing from newly born GCs is unknown.

To facilitate long-term tracking and visualization of posttrauma-born neurons, we used an inducible Cre/Lox transgenic reporter mouse. Cre recombinase was expressed in cells expressing Ascl1, a transcription factor that is transiently expressed in NPCs in the DG (Kim et al., 2007). Ascl1-CreERT2 mice were crossed with floxed tdTomato reporter mice to allow visualization of NPC development into mature GCs via indelible fluorescent dye labeling of the entire cytoarchitecture including the dendrites, cell soma and axon. Expression of tdTomato was induced in Ascl1-expressing NPCs during the first week after TBI and numbers, dendrite development and MFB synapses of fully matured GCs were evaluated at 6 weeks.

Pre-clinical TBI research has been conducted with a large male bias with ~93% of all studies using exclusively male mice (Spani et al., 2018). Despite evidence that adult neurogenic responses to external stimuli such as stress, exercise, and cognitive training differ for males and females (Chow et al., 2013; Hillerer et al., 2013; Yagi and Galea, 2019), few studies have examined proliferation or new neuron generation in females and males after TBI. While cellular proliferation after repeated mild TBI has been shown to be greater in the DG of adolescent males compared to females (Neale et al., 2023; Yamakawa et al., 2017), our data are the first to demonstrate sexual dimorphism in the development and survival of hippocampal neurons born after TBI. Additionally, we provide the new insights into injury-induced changes in adult newborn neuron synapse development, revealing sex-dependent responses that may have implications for hippocampal connectivity.

## 2 Methods

### 2.1 Transgenic mice

Heterozygous ^Ascl1 − CreRT2^ mice [Ascl1^∧^tm1(Cre/ERT2)Jejo/J; stock #012882] (Kim et al., 2007) and homozygous Ai14 mice [B6.Cg-Gt(ROSA)26Sortm14(CAG-tdTomato)Hze/J; stock #007914] (Madisen et al., 2010) obtained from The Jackson Laboratory (Bar Harbor, ME) were crossed to generate Ascl1-CreERT2; CAG-FloxStopTom mice. Mice were maintained on a C57Bl/6J background. Mice were provided with food and water *ad libitum* at the University of Kentucky Medical Center animal vivarium and were housed up to five mice per cage at a constant temperature (23 ± 2°C) with a 14/10 h light/dark cycle. All animal procedures were approved by the University of Kentucky's Institutional Animal Care and Use Committee under IACUC protocol 2019-3293.

Young adult (8–10 weeks of age) male and female mice were used except where noted. Mice were matched for age, rather than weight, since adult hippocampal neurogenesis is strongly regulated by age (Yang et al., 2015).

### 2.2 Controlled cortical impact and injections

For long-term neurogenesis studies, male and female Ascl1-CreERT2; CAG-FloxStopTom mice were assigned to CCI (*n* = 10 male, 9 female) or naive (*n* = 8 male, 11 female) groups. Surgeries were performed as previously described (Madathil et al., 2013). Briefly, the head of a mouse anesthetized using 3% isoflurane was secured in a stereotaxic frame. Anesthesia was maintained using 2.5% isoflurane delivered through a nose cone. A midline scalp incision was made and a 5 mm diameter craniectomy was performed over the left parietal cortex, lateral to the sagittal suture and midway between Bregma and Lambda. The exposed cortex was contused using a pneumatically controlled impacting device (TBI-0310, Precision Systems and Instrumentation, Lexington, KY) which drives a piston with a 3 mm diameter rounded tip at a rate of 3.5 m/s to a depth of 1.0 mm to produce a moderate injury. A moderate injury is defined here as cortical tissue loss in the absence of hippocampal ablation, leaving both pyramidal and GC layers grossly intact. After CCI injury, a circular disk made from dental cement was glued over the craniotomy to protect the brain surface and the scalp was closed. Mice received a 1 mL injection of saline subcutaneously for hydration and were placed on a heating pad to maintain body temperature until they recovered from anesthesia. Mice were then returned to their home cages. Naive mice did not undergo anesthesia or any surgical procedures.

Mice were injected intraperitoneally with 120 mg/kg BW tamoxifen (Sigma, St. Louis, MO) in corn oil on two consecutive days to induce Cre recombination. Naive mice were injected at 8 weeks of age. Injured mice were injected on days 2 and 3 after CCI to induce recombination in Ascl1-expressing cells born acutely after injury.

For examination of acute proliferation, a separate cohort of 9–11 week old male double transgenic reporter mice (*n* = 6 Ascl1-CreERT2; CAG-FloxStopTom) and tdTomato mice (*n* = 8 CAG-FloxStopTom) received CCI injury followed by tamoxifen injections as above. Mice were then injected intraperitoneally with 50 mg/kg of 5-ethynyl-2′-deoxyuridine (EdU; cat. A10044, Thermo Fisher Scientific, Waltham, MA) on days 3, 4, and 5 or days 6, 7, and 8 after injury. Mice were euthanized on day 10.

### 2.3 Tissue processing, histology and immunofluorescent labeling

Animals were euthanized at 10 days or 6 weeks after CCI or tamoxifen injection (naïve) by sodium pentobarbital (150 mg/kg i.p. Fatal-plus solution, Vortech Pharmaceuticals, LTD) and transcardially perfused with heparinized saline followed by 10% buffered formalin. Brains were removed from skulls 24 h after post-fixation in 10% formalin, further post-fixed for 24 h, and then cryoprotected using 30% sucrose solution. Brains were snap frozen in cold 2-methylbutane (< -25°C) and sectioned coronally at 40 μm thickness. Where possible, analyses were performed by individuals blinded to relevant independent variables such as sex, injury condition, genotype and time point of the samples. In many cases, it was not possible to be truly blinded to injury condition or hemisphere (ipsilateral/contralateral) due to obvious differences in the hippocampal formation ipsilateral to the CCI.

For analysis of cell proliferation, three sections containing dorsal hippocampus were selected at 400 μm intervals for each animal. Free-floating sections were reacted using a Click-iT^tm^ EdU Cell Proliferation Kit (cat. C10337, Thermo Fisher Scientific). TrueBlack (cat. 23007, Biotium, Fremont, CA) was then applied according to the manufacturer's instructions to quench autofluorescence. Slides were coverslipped with DAPI Fluoromount-G (Southern Biotech, Birmingham, AL) and stored at 4°C.

For contusion volume analysis, brain sections selected at 400 μm intervals spanning the rostral-caudal extent of the contusion were mounted on slides and stained for Nissl substance. A digital slide scanner (Zeiss Axio Sxan Z.1, Oberkochen, Germany) was used to image Nissl-stained sections at 20 × magnification to create a single high-resolution digital image. Halo software (version 2.3; Indica Labs, Albuquerque, NM) was used to analyze the images. For each section, the ipsilateral neocortical area containing surviving neurons, verified by examination of cell morphology, and the entire contralateral neocortical area were traced manually. Contusion area was calculated as the difference between the contralateral and spared ipsilateral neocortical areas. The contusion volume was then calculated using Cavalieri's principle.

Free-floating immunohistochemistry was performed to co-label tdTom+ neurons with the mature neuronal marker anti-NeuN (monoclonal mouse, 1:400, Novus NBP1-92693, St. Charles, MO) or the immature neuron marker anti-DCX (polyclonal rabbit, 1:2500, Abcam ab18723). Coronal sections were initially rinsed in TBS then blocked in 5% normal goat serum and 0.1% Triton-X-100 in TBS for 30 min at room temperature. Primary antibody was applied overnight at 4°C. The following day tissue was rinsed with TBS and incubated with Alexa Fluor 488 conjugated secondary antibody (1:500, Invitrogen, Carlsbad, CA) for 1 h at room temperature, and then rinsed with TBS. Sections were mounted on gelatin-coated slides which were then coverslipped with DAPI Fluoromount-G and stored at 4°C.

### 2.4 Cell counting and morphological assessments

#### 2.4.1 Manual counts of EdU+ cells

To assess acute cellular proliferation, the DG was imaged at 20 × magnification using an epifluorescent microscope (Ti2, Nikon, Melville, NY). Images taken as a z-stack with a 1.5 μm step size were stitched together to capture the entire length of the GC layer before conversion to a single MaxIP image. All images were analyzed in Image J. EdU labeled cells were manually counted within the SGZ and inner third of the GC layer. Counts were normalized to the length of the GC layer and averaged across sections for each animal.

#### 2.4.2 Manual counts of tdTom+ GCs

For manual counts of tdTomato+ (tdTom+) cells and Sholl analysis, three tissue sections per mouse selected at 400 μm intervals between −1.34 and −2.18 mm Bregma were mounted onto slides for analysis. Hippocampal sections were coverslipped with DAPI Fluoromount to visualize neuroanatomical structures within the DG. An epifluorescent microscope (Ti2, Nikon) was used for manual counting at 40 × magnification of tdTom+ GCs located in the upper and lower blades of the DG. Counts of GCs were normalized to the length of the GC layer by acquiring 10 × images of the DG using the DAPI signal. Images were imported into Fiji and the segmented line tool was used to outline and measure the length of the GC layer. Linear densities were averaged across three sections per animal.

#### 2.4.3 Sholl analysis

Images of tdTom+ hippocampal GCs were acquired as a z-stack (1.5 μm step size) at 20 × magnification using a confocal microscope (A1 Nikon, Minato City, Tokyo, Japan). For each animal, a total of six neurons in the upper blade of the GC layer were randomly selected across three tissue sections for manual digital tracings of tdTom+ dendrites using Fiji neuroanatomy Simple Neurite Tracer (SNT) plug in, an open-source software. Reconstructed dendritic arbors were analyzed for distance to the first branch, total length of dendrites and the number of dendritic intersections with a series of concentric circles at 10 μm intervals from the center of the cell body.

#### 2.4.4 Mossy fiber bouton counts and volumetric measurement

MFB volumetric measurement was conducted on two tissue sections containing dorsal hippocampus (−1.34 mm to −1.94 mm bregma). Images of tdTom+ pre-synaptic terminals in the CA3 region of the ipsilateral hippocampus were acquired as a z-stack (0.5 μm step size) at 40 × magnification using a Nikon A1 confocal microscope. Two image stacks from each animal from different tissue sections were imported into Imaris software (v.9.1, Bitplane AG, Zurich, Switzerland) for 3D visualization and reconstruction. Surface models of 3D image stacks (surface grain size 0.4 μm, threshold >480, seed point = 2.5) were created. Boutons that were cut by the boundaries, were not associated with an axon fiber, had volumes < 4 μm^3^ or had sphericity < 0.60 were not included in counts or volume measurements (LaSarge et al., 2015). Surface volume (μm^3^) measurements were exported to Excel and an average volume for each animal was calculated. The numbers of MFBs were summed across the two tissue sections for each animal. Numbers of boutons analyzed per group were 4,052 for male naïve, 779 for male CCI, 3,359 for female naïve, and 1,721 for female CCI.

### 2.5 Statistical analysis

Statistical analysis was conducted in either Prism or SAS. Counts of EdU+ cells, body weights and contusion volumes were analyzed with an unpaired two-tailed *t*-test with pooled variance. Counts of tdTom+ neurons were normalized to the length of the DG (mm). Normalized cell counts were log transformed due to right skewness and a mixed model was fit with a random effect to account for correlated observations within injured mice (ipsilateral and contralateral) and with fixed effects for sex, condition (CCI ipsilateral, CCI contralateral, and naïve), and the two-way interaction. Degrees of freedom were adjusted using the Kenward-Rogers adjustment. Sholl analysis data were analyzed using a 2-way repeated measures ANOVA with Fishers LSD for *post-hoc* comparisons. Length of primary dendrite and total dendrite length were analyzed using a 1-way ANOVA with Fishers LSD for *post-hoc* comparisons. MFB datasets were log transformed due to right skewness prior to conducting parametric 1-way ANOVAs. Parametric analysis of MFB parameters was corroborated using non-parametric Kruskal-Wallis ANOVAs; results were congruent except where noted in the Results. For 1-way ANOVA analyses only selected *post-hoc* tests were performed, as determined *a priori*; these included comparing sex within each injury condition and comparing injury condition within each sex. For contusion volume, measurements could not be completed on one female with CCI due to tissue tearing and folding. A subset of female naïve mice (*n* = 7–8) was randomly selected for Sholl tracing and MFB analyses. One male CCI mouse was excluded from the Sholl analysis and MFB volume measurements because it had no tdTom+ neurons in the DG or MFBs in the CA3 region of interest. Data are reported as mean and SEM. Graphs depict means and individual data points, with error bars representing SEM.

## 3 Results

### 3.1 Validation of transgenic reporter mouse model

**Figure 1:**
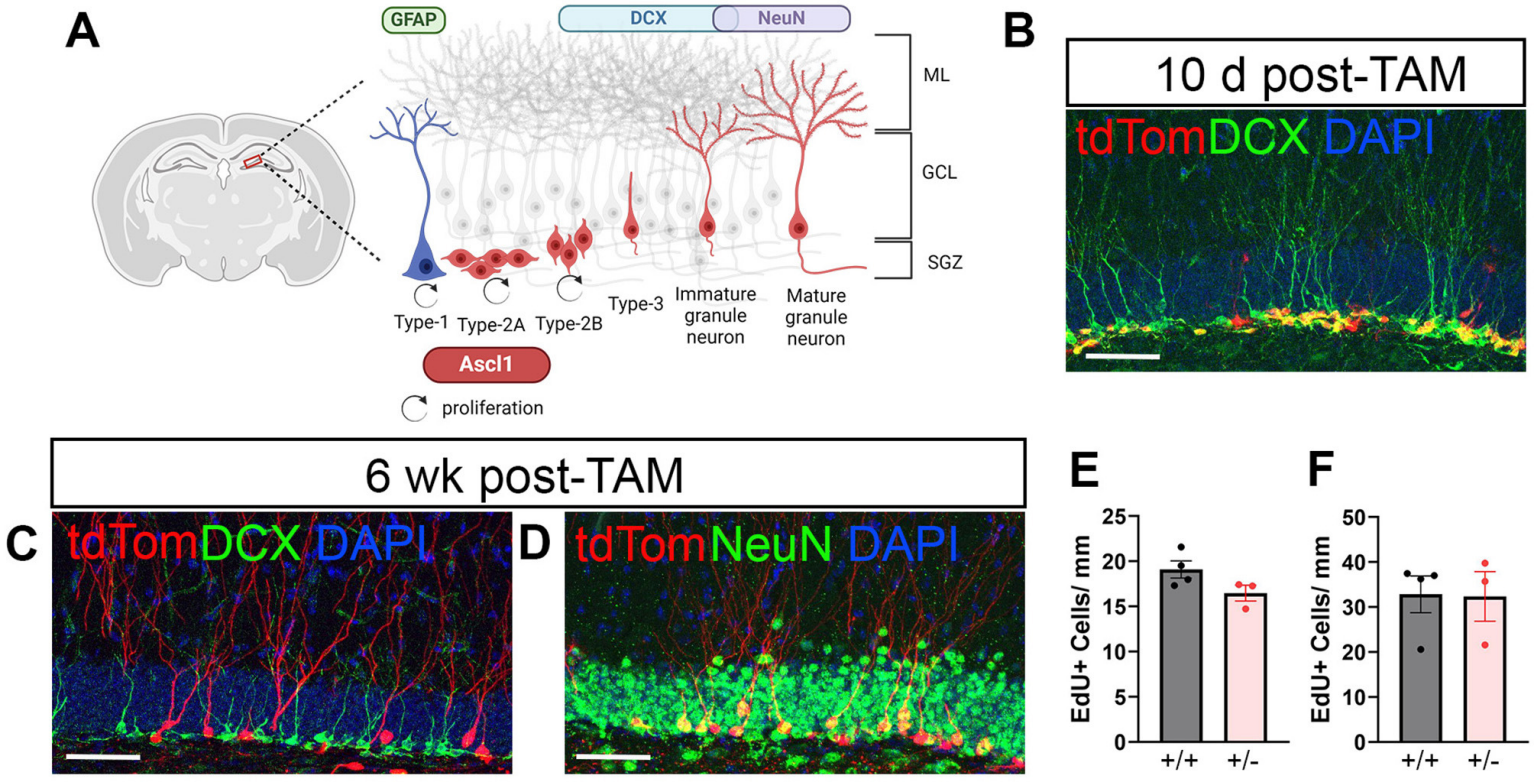
Neuronal maturation of Ascl1-derived neural progenitor cells (NPCs) in naïve mice. tdTomato (tdTom) conditionally induced in Ascl1-expressing NPCs allows visualization of granule cell (GC) development in the dentate gyrus during adult neurogenesis. **(A)** Diagram illustrating the transient expression of Ascl1 in early Type-2A cells and a subset of Type-1 cells in the adult dentate gyrus. ML, molecular layer; GCL, granule cell layer; SGZ, subgranular zone. **(B)** Ten days after tamoxifen (TAM) induction the majority of tdTom+ (red) cells co-labeled with doublecortin (DCX, green). **(C, D)** By 6 weeks after TAM induction tdTom+ cells had matured and no longer expressed DCX (green, in **C**) but co-labeled with the mature neuronal marker NeuN (green, in **D**). DAPI (blue). Scale bar = 50 μm. **(E, F)** Mice homozygous for Ascl1 (+/+) and transgenic reporter mice heterozygous for Ascl1 (+/–) have equivalent numbers of proliferating (EdU+) cells in the ipsilateral dentate gyrus during days 3–5 **(E)** and during days 6–8 **(F)** after CCI injury.

To genetically label NPCs with a fluorescent dye, tamoxifen-inducible Ascl1-CreERT2; CAG-FloxStopTom mice were used. Ascl1 is a proneural transcription factor that is transiently expressed in a subset of NPCs in the peripheral and central nervous system (Guillemot and Joyner, 1993; Wapinski et al., 2013). In the hippocampal DG of adult mice, the majority of cells expressing Ascl1 are Type-2A early NPCs (Kim et al., 2007). Ascl1 expression is downregulated as the cell commits to a neuronal fate, designated as Type-2B (Figure 1A). A subset of Type-1 activated NSCs express Ascl1 (Andersen et al., 2014; Kim et al., 2007) and can be identified by co-labeling with GFAP.

Ten days after tamoxifen induction in naïve mice, the majority of tdTom+ cells in the DG phenotype as neuroblasts or immature neurons as defined by their expression of the microtubule-associated protein doublecortin (DCX) (Figure 1B). As adult-born neurons mature, they elaborate dendritic arbors, downregulate expression of DCX and express the mature neuronal marker NeuN. Accordingly, at 6 weeks after inducing Cre-recombination in Ascl1 expressing NPCs, tdTom+ cells were no longer DCX+ (Figure 1C) but co-labeled with NeuN (Figure 1D). Retention of fluorescent dye in cells as they developed into fully mature GCs allowed visualization and assessment of the cells' morphological development of dendrites as well as their axonal projections to the CA3 region.

Because the Ascl1-CreERT2 mouse line used here has only one functioning Ascl1 allele (Kim et al., 2007) and Ascl1 is required for NSC/NPC activation, we compared cellular proliferation in Ascl1^+/+^ mice (CAG-FloxStopTom mice) and Ascl^+/−^ mice (Ascl1-CreERT2; CAG-FloxStopTom). Following EdU injections on days 3, 4, and 5 postinjury to capture cells proliferating early after tamoxifen induction, no difference was noted in numbers of EdU+ cells in the SGZ and inner third of the GC layer of the ipsilateral hippocampus at 10 days following injury (Figure 1E). Similarly, proliferation on days 6, 7, and 8 after injury was equivalent in the two groups of mice (Figure 1F). This suggests that the Ascl1 heterozygosity of the double transgenic reporter mice did not influence cellular proliferation within the neurogenic niche across the first week after CCI.

### 3.2 Cortical contusion volume is equivalent in age-matched male and female mice

**Figure 2:**
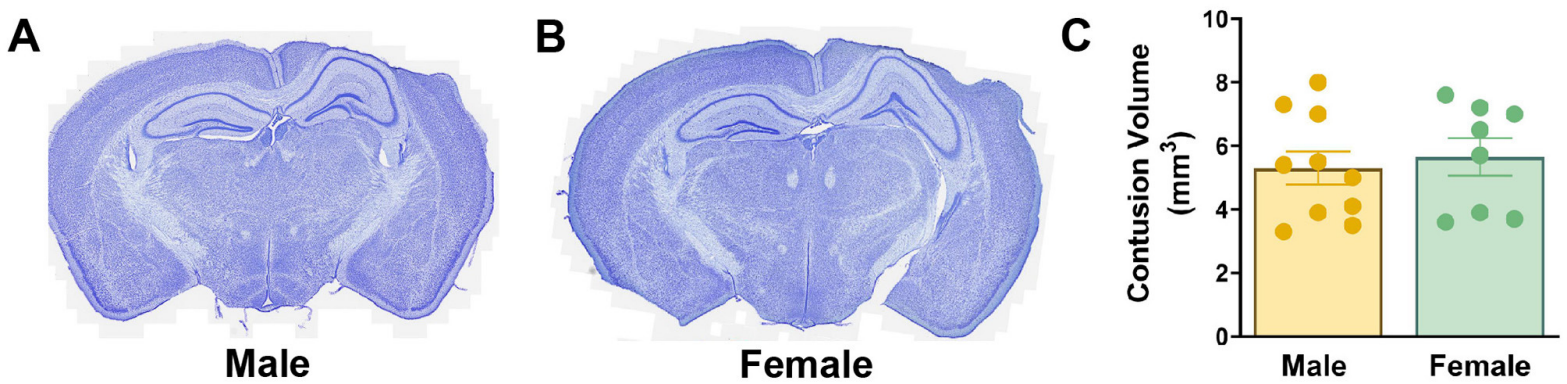
Cortical tissue loss is equivalent in age-matched male and female mice following CCI. **(A, B)** Nissl-stained coronal sections of male **(A)** and female **(B)** CCI-injured mice. **(C)** Quantification of cortical contusion volume at 6 weeks after injury.

CCI is a widely used model of contusive brain injury resulting in a progressive cortical neuron death within a hemorrhagic contusion leading to the formation of a cavity (Williams et al., 2022). We sought to determine whether age-matched male and female mice might have different degrees of cortical damage, which could potentially confound interpretation of neurogenic responses as the extent of hippocampal neurogenesis is dependent on injury severity (Wang et al., 2016). At 8–10 weeks of age, female mice weighed significantly less (20.7 ± 1.0 g) than male mice (25.8 ± 1.9 g) at the time of injury (*p* < 0.0001). Nonetheless, 3 days after injury males and females exhibited similar weight gain (males 0.8 ± 0.8 g, females 0.8 ± 1.1 g). Pathological features in injured males (Figure 2A) and females (Figure 2B) were typical of moderate severity CCI. There was extensive neocortical tissue loss and cavitation ipsilateral to impact while the underlying hippocampal formation remained grossly intact, with no ablation of the pyramidal or GC layers. Using the Cavalieri method, volumetric estimates of the contusion within the neocortex were performed. Age-matched male and female mice had equivalent cortical contusion volumes 6 weeks after CCI injury (Figure 2C).

### 3.3 Females show a greater neurogenic response to CCI compared to males

**Figure 3:**
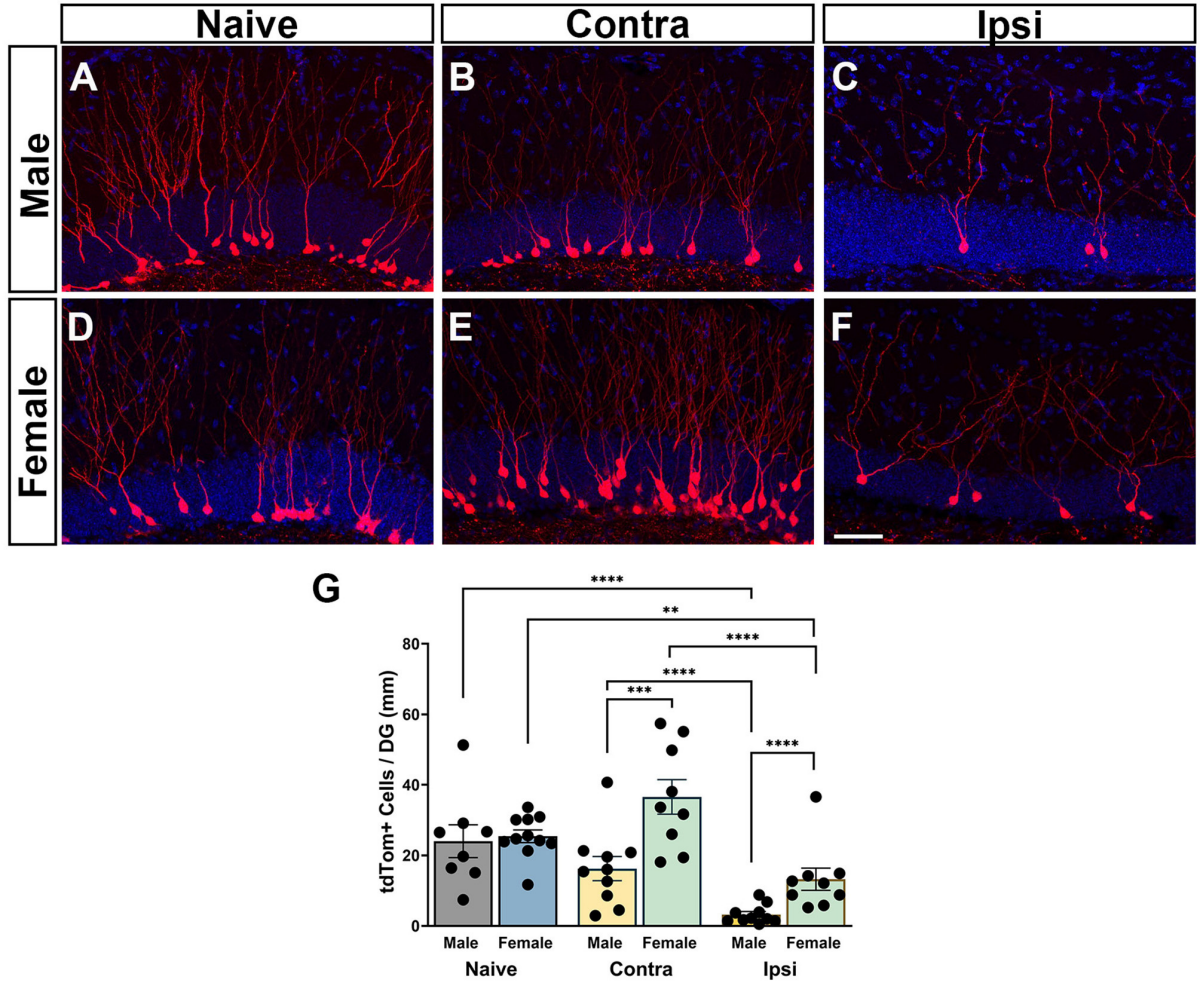
Regional neurogenic responses to TBI are sex-dependent. **(A, D)** Adult-born granule neurons in naïve male and female mice visualized by tdTomato expression (red) at 6 weeks after tamoxifen administration. **(B, C, E, F)** CCI injury results in a deficit in neurogenesis in the ipsilateral dentate gyrus **(C, F)** as compared to the contralateral dentate gyrus **(B, E)** with fewer posttrauma-born neurons at 6 weeks after injury in male and female mice. DAPI = blue. Scale bar = 50 μm. **(G)** Counts of tdTom+ neurons demonstrate that injured females have more surviving posttrauma-born neurons than injured males in both the ipsilateral and contralateral hippocampus. ^**^p < 0.01, ^***^p < 0.005, ^****^p < 0.0001.

To examine how sex influences numbers of GCs born acutely after injury that survive to maturity, the number of tdTom+ neurons in the DG GC layer was quantified in male and female naïve mice and CCI-injured mice at 6 weeks after injury. In naïve mice, numerous tdTom+ neurons were localized in the inner one third of the GC layer (Figures 3A, D). Labeled adult-born neurons had round cell somas with well-developed dendritic arbors extending through the molecular layer. In CCI-injured mice, posttrauma-born neurons were less abundant in the ipsilateral DG in both males and females. Their somas were localized within the GC layer in both the contralateral and ipsilateral DG, although neurons that had migrated to the outer two-thirds of the GC layer were more frequently observed than in naïve mice (Figures 3B, C, E, F). This deeper migration was most notable in the contralateral DG of female mice, which also showed more abundant tdTom+ neurons (Figure 3E). Dendrites of posttrauma-born neurons could be visualized extending well into the molecular layer.

Quantification of numbers of tdTom+ neurons revealed a main effect of sex [*F*_(1, 33.8)_ = 21.87; *p* < 0.0001] with females demonstrating increased neuron numbers. There was a main effect of the condition [*F*_(2, 27.7)_ = 87.09; *p* < 0.0001] and an interaction of sex and condition [*F*_(2, 27.7)_ = 7.42; *p* = 0.0026]. *Post-hoc* analyses revealed that both male and female CCI-injured mice had significantly fewer tdTom+ neurons in the ipsilateral hippocampus (Figures 3C, F) compared either to naïve controls (Figures 3A, D) (males *p* < 0.0001, females *p* < 0.0067) or to cell counts in the contralateral hippocampus (Figures 3B, E) (males *p* < 0.0001, females *p* < 0.0001), consistent with a trauma-induced decrease in neurogenesis in the ipsilateral DG. Males showed a more pronounced deficit in posttraumatic neurogenesis, with significantly fewer tdTom+ cells in the ipsilateral hippocampus compared to females (*p* < 0.0001; Figure 3G). Neurogenesis in the contralateral hippocampus of CCI-injured mice was not different from that in naïve mice for either males or females. However, the number of tdTom+ neurons in the contralateral hippocampus of CCI-injured females was significantly greater than the number in the contralateral hippocampus of CCI-injured males (*p* = 0.0013; Figure 3G), pointing to sexually dimorphic neurogenic responses in the contralateral as well as the ipsilateral hippocampus.

### 3.4 Males demonstrate impairments in posttrauma-born neuron dendritic arbor morphology not observed in females

**Figure 4:**
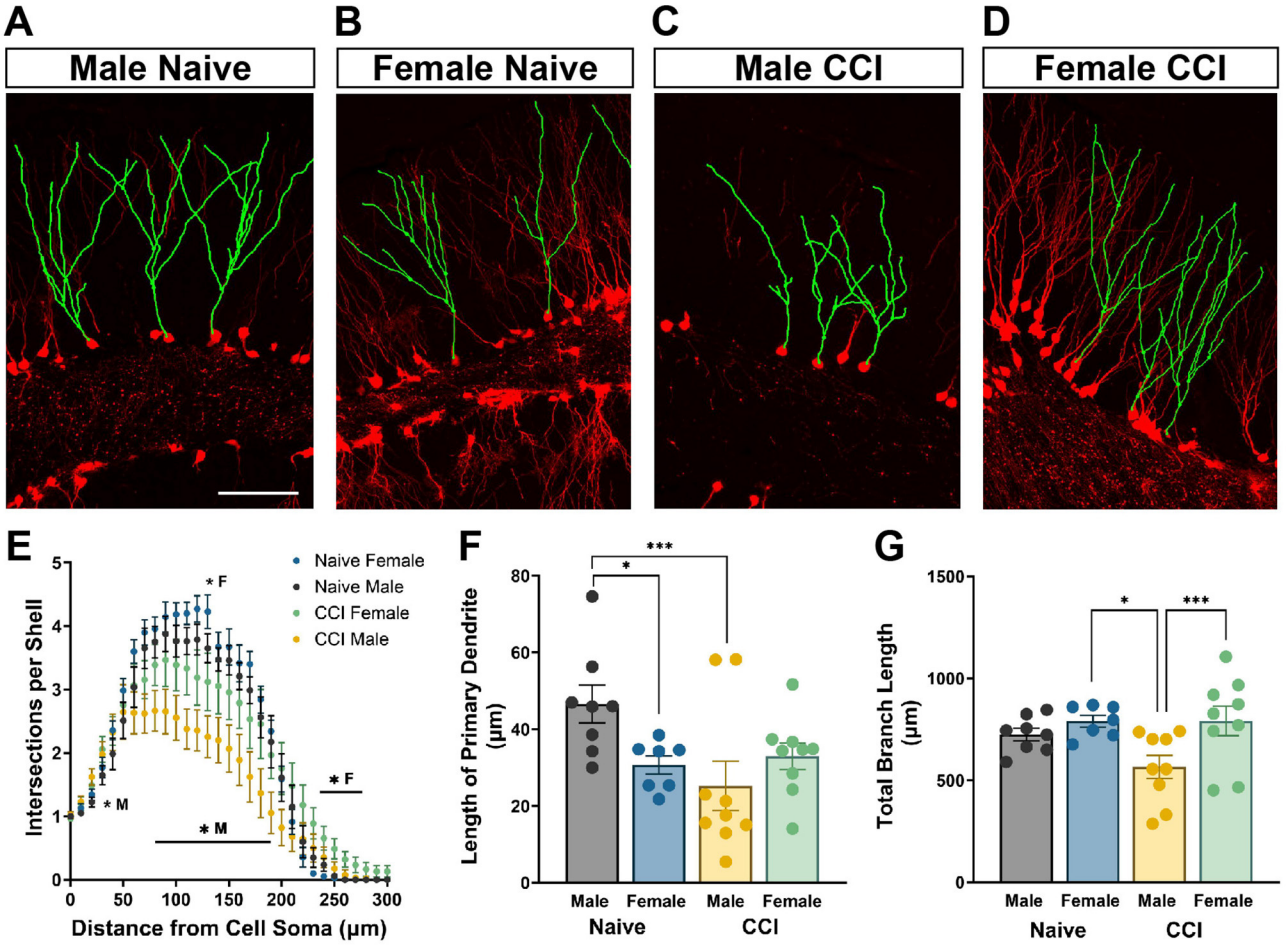
Neurons born after injury in male mice demonstrate impairments in dendritic arbor morphology not observed in posttrauma-born neurons in female mice. Representative images demonstrating tdTom+ (red) dendrites digitally traced (green) and reconstructed for Sholl analysis of **(A)** male naïve, **(B)** female naïve, **(C)** male CCI-injured, and **(D)** female CCI-injured mice. Scale bar = 100 μm. **(E)** CCI-injured males show a greater decrease in dendritic complexity than CCI-injured females. ^*^M: CCI-injured males different than naïve males, ^*^F: CCI-injured females different than naïve females. **(F)** Posttrauma-born granule neurons in male mice develop with shorter primary dendrites than adult-born neurons in male naïve mice. **(G)** The total dendritic branch length of tdTom+ labeled GCs in CCI-injured males is significantly decreased compared to CCI-injured females and to naïve male controls. ^*^p < 0.05, ^***^p < 0.005.

tdTom+ GCs in naïve mice and in the contralateral DG of injured mice displayed an apical dendrite that extended through the GC layer and initiated branching in the inner molecular layer (Figures 4A, B). However, dendrites of tdTom+ GCs in ipsilateral DG of injured mice often began branching within the GC layer (Figures 4C, D; see also Figure 3). To examine how TBI influences the development of dendritic arbors of GCs born acutely after injury that survive to maturity, tdTom+ neurons in the upper blade of the GC layer were digitally traced and reconstructed for Sholl analysis. Dendritic complexity did not differ between male and female naïve mice [*F*_(30, 390)_ = 0.8615; *p* = 0.6796] (Figure 4E). Compared to same sex naïve controls, both male and female CCI-injured mice had significantly altered dendrite complexity [male: *F*_(30, 450)_ = 4.876; *p* < 0.0001, female: *F*_(30, 420)_ = 3.492; *p* < 0.0001]. Although there was no significant difference in dendrite complexity between male and female CCI-injured mice when comparing across the entire length of the arbor [*F*_(30, 480)_ = 1.307; *p* = 0.1308], the Sholl analysis curves suggested unique changes for males and females. In injured males, the Sholl curve showed a more marked downward shift across distances between 70 and 200 μm from the soma (Figure 4E). Branching was significantly less from 80 to 190 μm from the cell soma while in CCI-injured female mice branching was only significantly reduced at 130 μm. Conversely, dendritic branching was increased significantly increased at 230–270 μm from the cell soma in female mice and at 30 μm in male mice.

Adult-born GCs in naïve females had a significantly shorter distance to the first branch point than those in naïve males (*p* < 0.05; Figure 4F) although the total dendritic length of all dendrite branches was comparable (Figure 4G). In CCI-injured male mice the primary dendrite was significantly shorter compared to naïve males [*F*_(3, 29)_ = 3.623; *p* = 0.0246] (*p* = 0.003). In contrast, CCI-injured females had no change in length of the primary dendrite (Figure 4F). Similarly, posttrauma-born GCs in males developed with dendritic arbors of significantly decreased total length compared to GCs born in naïve males [*F*_(3, 29)_ = 4.022; *p* = 0.0165] (*p* = 0.044, Figure 4G), while branch length was unaffected by CCI in females. Further, GCs born after injury in females had significantly greater total dendritic length than those in males (*p* = 0.0046).

### 3.5 Posttrauma-born neurons extend axons to CA3 and form synapses in a sex-dependent manner

**Figure 5:**
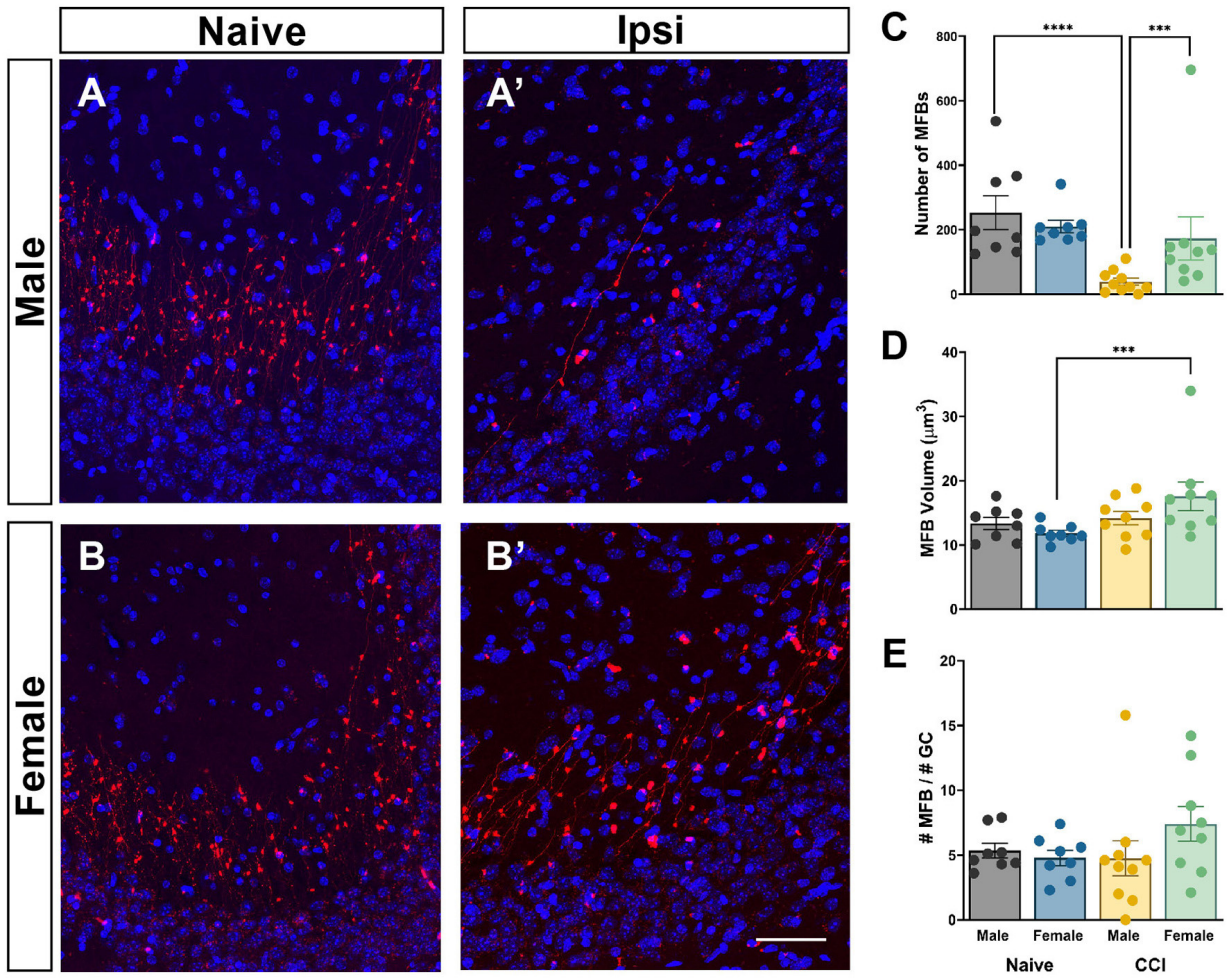
Mossy fiber boutons are altered after CCI in a sex-dependent manner. Representative images demonstrating tdTom+ (red) pre-synaptic terminals in the CA3 region of **(A)** naïve male, **(B)** naïve female, **(A')** CCI-injured male, and **(B')** CCI-injured female mice. DAPI = blue. Scale bar = 10 μm. **(C)** The average number of mossy fiber boutons (MFBs) is significantly less in CCI-injured males than in CCI-injured females or in male naïve controls. **(D)** The average surface volume of MFBs is significantly increased only in CCI-injured female mice. **(E)** Numbers of MFBs per GC did not differ significantly across groups. ^***^p < 0.005, ^****^p < 0.0001.

To determine if TBI alters MFB number or volume in the CA3 region, MFBs were reconstructed in 3D from high resolution confocal images (Figures 5A, A', B, B'). Far fewer tdTomato+ MFBs were visible in the hippocampus of male mice with CCI when compared to all other groups. Naïve mice demonstrated no sex-dependent differences in numbers of MFBs in the CA3 region, MFB surface volume, or the relative ratio of numbers of MFB to numbers of tdTom+ GC (Figures 5C–E). Numbers of MFBs varied significantly across groups [*F*_(3, 30)_ = 14.91; *p* < 0.0001]. *Post-hoc* tests revealed numbers of MFB in the CA3 region of male mice with CCI was significantly reduced to 39 ± 11 from 253 ± 53 in naïve controls (*p* < 0.0001) (Figure 5C). Numbers of MFBs in female mice with CCI (173 ± 67) were somewhat lower than in naïve controls (210 ± 20). This decrease was not statistically significant by ANOVA after log transformation (*p* = 0.132) but was significant using nonparametric analysis (*p* = 0.049). Brain-injured females had significantly greater numbers of MFBs from posttrauma-born neurons than did brain-injured males (*p* = 0.0002). Only CCI-injured female mice displayed increased MFB surface volume (17.6 ± 2.2 μm^3^) when compared to sex-matched naïve controls (11.8 ± 0.49 μm^3^) (*p* = 0.004) [*F*_(3, 30)_ = 3.436; *p* = 0.0293] (Figure 5D). The average MFB surface volume in male mice was unchanged after CCI. To determine if the decrease in MFB numbers in the CA3 region was proportional to the decrease in tdTom+ GCs in the DG after TBI, we normalized the number of MFB to the number of tdTom+ GCs for each mouse. Despite a >50% increase in MFB numbers per GC in CCI-injured females (7.40 ± 1.34) compared to males (4.75 ± 1.36), the numbers of MFB per GC did not vary statistically across groups [*F*_(3, 30)_ = 1.057; *p* = 0.3820] (Figure 5E).

## 4 Discussion

Conditional induction of tdTomato expression in Ascl1-expressing Type-2A NPCs allows for visualization of GC development in the DG during adult neurogenesis. Using this model, we confirmed differentiation of Ascl1-expressing NPCs to DCX+ neuroblasts and immature neurons by 10 days after induction and maturation to NeuN+ neurons by 6 weeks. Male and female naïve mice generated equivalent numbers of adult-born GCs with similar morphological development of dendrites and numbers of MFBs. The only notable sex-related difference was that GCs born in female naïve mice exhibited a shorter primary dendrite.

Using the Ascl1-tdTomato transgenic model, we demonstrate that in both male and female mice TBI causes a profound decrease in numbers of GCs born in the first week after injury that survive to 6 weeks. Many have shown that early loss of immature neurons in the hippocampal DG after CCI is accompanied by a transient increase in cellular proliferation within the SGZ that supports the recovery of the immature neuron population through neurogenesis (Kernie and Parent, 2010; Bielefeld et al., 2019). However, whether this wave of proliferation supports a net gain in mature GCs is less clear. Tracking the survival and maturation of birth-dated GCs is most commonly achieved either through administration of BrdU or its analogs to label proliferating cells or by conditional induction of reporter molecule expression selectively in neural stem cells, NPCs or immature neurons. By labeling cells born during the first week after TBI and quantifying the number that colabel with a mature neuron marker such as NeuN at 1 month or later, investigators have reported increased (Kernie et al., 2001; Villasana et al., 2015; Yu et al., 2008; Han et al., 2011), unchanged (Gao and Chen, 2013; Littlejohn et al., 2020; Rice et al., 2003), or decreased (Kang et al., 2022; Rola et al., 2006) neurogenesis. These studies used either exclusively male mice or reported results pooled for males and females.

Disparate findings regarding the effect of TBI on generation of mature neurons may be due in part to differences in injury model, location or severity. As an example, hippocampal neurogenesis has been shown to increase after severe, but not moderate or mild, CCI (Wang et al., 2016). In addition, differences in dose, timing and frequency of BrdU injections or the promoter used to elicit reporter expression make comparisons across studies challenging. As we chose naïve mice as our control group, we cannot rule out that the craniotomy procedure or isoflurane exposure may have contributed to decreased neurogenesis observed in CCI-injured mice. Although this has not been well-studied, a craniectomy performed under ketamine/xylazine anesthesia was found to increase, rather than decrease, numbers of immature neurons in the ipsilateral hippocampus (Aleem et al., 2020). Further, studies of 2–4 h of isoflurane exposure, a much longer duration than used with CCI, show modest (Kim et al., 2020) or no (Bockmann et al., 2023) effects on acute proliferation and no effect on numbers of newly born immature or mature neurons.

We selected an Ascl1 reporter construct in order to label activated NSCs/NPCs, effectively birth-dating cells at an early stage of the neurogenic process. Expression of the proneural protein Ascl1 is required for Type-1 radial glia-like (RGL) NSCs to exit quiescence and become activated in response to external stimuli. Thus, conditional deletion of both Ascl1 alleles in adult mice prevents activation of RGL cells (Andersen et al., 2014). The effect of Ascl1 heterozygosity on RGL activation is not known, but because the Ascl1-Cre mice used here have only one functioning Ascl1 allele (Kim et al., 2007) and TBI activates RGL cells to stimulate the neurogenic response (Gao et al., 2009; Yu et al., 2008), it is possible that numbers of posttrauma-born cells born in response to TBI in Ascl1-tdTomato transgenic mice were blunted. However, numbers of proliferating cells in the SGZ and inner GC layer in brain-injured Ascl1-tdTomato transgenic mice were equivalent to those in tdTomato mice possessing two Ascl1 alleles. Later stages of neurogenesis should not be affected by Ascl1 heterozygosity as Ascl1 is not expressed in neuroblasts or immature neurons (Andersen et al., 2014). The ability of both naïve and CCI-injured Ascl1-Cre mice to generate mature GCs from NPCs suggests one functioning allele for Ascl1 is sufficient for hippocampal neurogenesis.

While both males and females showed decreased neurogenesis after CCI, we found notable sex differences in neurogenic responses, with injured males generating significantly fewer mature, posttrauma-born GCs. To our knowledge, only two other studies in adult rodents have compared sexes with respect to posttraumatic hippocampal neurogenesis. Gomez-Porcuna et al. (2024) examined only the contralateral hippocampus, reporting no CCI-induced change in immature GC numbers in male or female rats. Xiong et al. (2007) employed a severe CCI associated with loss or extensive thinning of the upper GC layer and found that numbers of acutely proliferated cells in the DG and the percentage that phenotyped as neurons at 35 days postinjury did not differ for male and female mice when using a broad window, 10-day BrdU injection paradigm (Xiong et al., 2007). Our finding of fewer surviving posttrauma-born GCs in males using a brief tamoxifen induction at days 2–3 may point to a critical window for sex-dependent effects on proliferation restricted to the first days after injury. Alternatively, sex-dependent regulation of neurogenic responses may be severity dependent and more evident in our moderate CCI paradigm.

The process of neurogenesis depends upon regulation of NPC proliferation as well as newborn neuron maturation and survival. Although each of these neurogenic stages could be sexually dimorphic, we cannot ascertain their relative roles in the current experimental construct. In naïve mice, male rats have higher levels of cell proliferation, faster newborn neuron maturation, but greater attrition of new neurons compared to females, yielding an equivalent net production of mature, adult-born GCs (Yagi et al., 2020). A heightened neuroinflammatory response after CCI injury in male mice (Doran et al., 2019; Villapol et al., 2017) could contribute to reduced neurogenesis, as activated microglia can suppress proliferation and increase attrition of adult-born neurons (Ekdahl et al., 2009; Navabi et al., 2024).

After TBI, immature hippocampal neurons exhibit decreased dendritic length and complexity (Carlson et al., 2014; Hood et al., 2018; Shahror et al., 2020) and early, increased proximal dendrite branching (Villasana et al., 2015; Hung et al., 2023), abnormalities that persist to maturity (Ibrahim et al., 2016; Villasana et al., 2015). However, sex-specific characteristics of dendrite development have not been reported. Here, we show that while granule neurons born after injury in male mice matured with dendritic arbors characterized by decreased complexity, reduced total branch length and a shorter proximal dendrite, posttrauma-born neurons in female mice developed with dendritic arbors nearly equivalent to those in naïve females. Improved dendrite development in neurons born after TBI in females may enhance synaptic communication between the entorhinal cortex and DG.

To our understanding, this is the first study to quantify TBI-induced structural changes in the synaptic terminals of posttrauma-born neurons. CCI-injured males exhibited a highly significant reduction in the numbers of tdTom+ MFBs in the CA3 region, while injured females showed only a modest reduction. Indeed, female mice maintained significantly more MFBs at 6 weeks after injury than did male mice. The loss of MFBs in male mice may be primarily due to reduced numbers of posttrauma-born neurons that survive to 6 weeks, as the ratio of MFBs to GC numbers was not significantly reduced from naïve mice. Although not statistically significant, CCI-injured females showed a more than 50% increase in MFBs per GC compared to CCI-injured males, raising the possibility of injury-induced changes in synaptic pruning or synaptogenesis. Synaptic adaptation in the form of increased surface volume was only observed in females. A reduction in bouton density can be offset by a compensatory increase in the number of release sites on individual MFBs, which can be measured indirectly through surface volume (LaSarge et al., 2015). Thus, injured females may compensate for decreased synaptic input resulting from lower numbers of posttrauma-born neurons in part by increasing neurotransmitter release sites. The functional consequences of sex-dependent neurogenic responses are not yet known. Electrophysiological and behavioral measures could be incorporated into future studies to interrogate the functional consequences of sexually dimorphic dendrite and synapse development of posttrauma-born neurons. Several studies suggest CCI-injured male and female rodents have equivalent object recognition memory and visuospatial learning and memory function assessed in a Morris water maze (Tucker et al., 2016; Wagner et al., 2004; Xiong et al., 2007; Gomez-Porcuna et al., 2024), but further testing using neurogenesis-dependent cognitive assays such as reversal learning or place or pattern recognition is needed.

Female mice responded to TBI with greater structural synaptic plasticity in the ipsilateral DG as well as a significant increase in numbers of new neurons born in the contralateral DG not observed in male CCI-injured mice. While these responses may be adaptive, they could constitute aberrant neurogenesis. Increased MFB volume or number may result in increased neurotransmitter release or divergence onto CA3 pyramidal cells, factors linked to the development of limbic epilepsy (Danzer et al., 2010). CCI-injured rodents that develop post-traumatic epilepsy (PTE) have increased numbers of ectopically localized immature neurons, loss of hilar interneurons, and mossy fiber sprouting in the DG and CA3 regions (Gudenschwager-Basso et al., 2023; Hunt et al., 2009; Sun et al., 2022). While increased MFB plasticity in females following CCI could point to an increased risk for developing limbic epilepsy, females have been reported to have a somewhat lower incidence of seizures after CCI (Kochanek et al., 2006). We did not observe aberrant mossy fiber sprouting in the DG molecular layer in either sex at 6 weeks after injury, but longer survival times should be evaluated to determine whether females have a higher propensity for mossy fiber sprouting in patterns typical of posttraumatic epilepsy.

In conclusion, at 6 weeks following CCI injury, both male and female mice demonstrated decreased neurogenesis in the ipsilateral DG. Neurogenic impairment was more profound in males, with significantly fewer posttrauma-born neurons, less developed dendritic arbors and fewer MFBs in the CA3 region compared to females. In contrast, GCs born acutely after injury in females developed with near normal dendritic arbors. CCI-injured females also showed potential compensatory responses such as enhanced contralateral hippocampal neurogenesis and increased MFB volume. Additional studies investigating mechanisms underlying these sexually dimorphic responses as well as their functional implications are warranted. Our findings emphasize a critical need to incorporate sex as an independent physiological variable in studies of therapeutic interventions targeting posttraumatic hippocampal neurogenesis in order to alleviate cognitive dysfunction after TBI.

## Data Availability

The original contributions presented in the study are included in the article/supplementary material. Further inquiries can be directed to the corresponding author.
